# Redox Metabolism and Autonomic Regulation During Aging: Can Heart Rate Variability Be Used to Monitor Healthy Longevity?

**DOI:** 10.3390/biomedicines13010161

**Published:** 2025-01-10

**Authors:** Olha Yelisyeyeva, Danylo Kaminskyy, Marta Semen, Ilona Chelpanova, Khrystyna O. Semen

**Affiliations:** 1 Department of Histology, Cytology and Embryology, Danylo Halytsky Lviv National Medical University, 79010 Lviv, Ukraine; yelisol@gmail.com (O.Y.); ilona.med75@gmail.com (I.C.); 2 Department of Pharmaceutical, Organic and Bioorganic Chemistry, Danylo Halytsky Lviv National Medical University, 79010 Lviv, Ukraine; dankaminskyy@gmail.com; 3 Department of Propaedeutics of Pediatrics and Medical Genetics, Danylo Halytsky Lviv National Medical University, 79010 Lviv, Ukraine; martasemen4@gmail.com; 4 Campus Venlo, Maastricht University, 5911 BV Venlo, The Netherlands

**Keywords:** aging, anti-aging, heart rate variability, hormetic response

## Abstract

The functionality of redox metabolism is frequently named as an important contributor to the processes of aging and anti-aging. Excessive activation of free radical reactions accompanied by the inability of the antioxidant defense (AOD) mechanisms to control the flow of the reactive oxygen species (ROS) leads to the persistence of oxidative stress, hypoxia, impaired mitochondrial energy function and reduced ATP potential. From a long-term perspective, such changes contribute to the development of chronic diseases and facilitate aging. In turn, preconditioning of a biosystem with small doses of stressful stimuli might cause mobilization of the mechanisms of AOD and control an excessive flow of ROS, which supports optimal functioning of the redox reactions. Those mechanisms are of crucial importance for anti-aging and are also known as a eustress or hormetic response. To ensure continuous support of mild pro-oxidant activity in a metabolic system, close monitoring and timely corrections preventing the development of excessive ROS production are required. The paper introduces the potential of heart rate variability (HRV) as a biomarker of functional and metabolic reserves and a tool to measure stress resilience during aging. The practical approaches to interpretation of HRV are provided based on total power, changes in total power in response to an orthostatic test and activities of all spectral components. It is suggested that the complex of those parameters can reflect the depth of oxidative stress and may be used to guide lifestyle interventions and promote active longevity.

## 1. Introduction

The problem of aging gained increased significance over the past two centuries, primarily due to major advances in biomedical sciences and improved medical care, which allowed humanity to significantly extend life expectancy [1,2,3]. Nowadays, late-life diseases, such as cancer, type 2 diabetes mellitus, and cardiovascular and neurodegenerative diseases are recognized as common causes of reduced quality of life and increased rates of hospital referrals worldwide, presenting a substantial burden on healthcare systems [4,5,6]. Extensive research was performed to clarify specific causes and mechanisms involved in aging [6,7] and several theories of aging were introduced highlighting the importance of oxidative damage, genetic mutations, accumulation of waste products, changes in circadian regulation (“wear and tear”) and development of neuroendocrine and autoimmune disorders [8,9,10,11]. At the same time, the complexity of pathological processes involved in aging hampers the identification of the optimal biomarkers that can be used to follow its progression and/or monitor the effects of anti-aging therapies.

This review aims to analyze the potential role of heart rate variability (HRV) as an integrative biomarker that can be used to monitor healthy aging. The role of reactive oxygen species (ROS) in the mechanisms involved in aging and anti-aging is discussed. The evidence suggesting the links between redox metabolism and autonomic regulation is introduced and the practical approaches to interpreting HRV as a biomarker of oxidative stress and a promising tool guiding the interventions promoting healthy longevity are given.

## 2. Pathological Versus Physiological Role of Free Radicals in Aging

In 1956, Denham Harman proposed the Free Radical Theory of aging, which remains widely accepted due to its robust interpretation of the molecular and cellular changes associated with aging [12,13,14,15]. Based on the evidence available at that time, this theory emphasized the harmful role of free radicals. It recognized that both excessive production of reactive oxygen species (ROS) and inefficient mechanisms of antioxidant defense contribute to impaired cell functionality, particularly because of the deleterious effects of ROS on the membranous structures [16,17,18]. Subsequently, the mechanisms leading to progressive failure to maintain a balance between pro-oxidants and antioxidants were systematized by Helmut Sies into the concept of oxidative stress, which continues to be extensively researched and debated [17,19,20]. The hallmarks of oxidative stress include accumulation of oxidative destruction products, development of cellular hypoxia and reduction in the speed of the electron transfer in the mitochondrial electron transport chain [21,22]. The latter facilitates the uncontrolled release of ROS by the mitochondria and promotes a significant energy deficit which further compromises regulatory mechanisms aiming to balance ROS production and utilization and achieve an optimal stimulation of antioxidant defense. Importantly, increased ROS flow has a major impact on the mechanisms of redox signaling, further contributing to the persistence of the signs of oxidative stress, chronic inflammation [16,20,23], and delay in the regeneration of damaged functional structures. The persistence of such a metabolic situation is recognized as one of the main causes of accelerated aging [23]. Moreover, it has been implicated in the pathobiology of many age-related chronic diseases [4,24,25,26].

Furthermore, as early as the 1980s, it was demonstrated that the production of free radicals is essential for cell functionality due to their role in proliferation, differentiation and signal transduction [27,28,29]. Various ROS including superoxide, hydrogen peroxide, and nitric oxide are recognized to be physiological intracellular metabolites involved in cell signaling and maintenance of redox homeostasis [30,31,32,33]. Furthermore, the flow of ROS was documented to be a critical modulator of the activity of the transcriptional factors such as NF-кB, Nrf2, and HIF-1α, which regulate the expression of genes involved in responses to oxidative stress, hypoxia, and inflammation and ensure reciprocal control of oxidative homeostasis [23,29,34,35]. The bioenergetic function of the mitochondria is closely linked to the rate of free radical production and, thus, largely defines the activity of redox reactions and responses of the antioxidant defense system to the oxidative load [28,36,37]. It has been postulated that some optimal levels of cellular ROS are essential for maintaining redox homeostasis and developing adaptive responses and functional improvements in response to various challenges. Triggering of adaptation (eustress) involves mild shifts in the pro-/antioxidant balance to the left, leading to the stimulation of mitochondrial function and aerobic metabolism. Interestingly, the same mechanisms have been reported to play a role in interventions aimed at promoting longevity [28,38,39,40,41,42], suggesting that optimal modulation of the ROS flow is important for healthy aging.

## 3. Hormetic Response as a Way to Promote Anti-Aging

The capacity to develop and maintain a hormetic response was demonstrated to be predictive of the duration of healthy active life span [3,43,44,45]. The hormetic response (eustress) is initiated by the upregulation of the pathways, which are also involved in the development of oxidative stress. However, it entails distinct principles of organization of the key molecular and cellular processes, eventually leading to better control over the production of free radicals and improved adaptation [45]. Initially, regular exposure to adaptive stressors (hormetic factors) activates fast but low-amplitude fluctuations in the ROS flow, which interacts with various components of the endomembrane system. This causes moderate activation of the enzymatic antioxidant defense system and promotes the elimination of oxidatively damaged macromolecules [29,38]. Induced moderate energy deficit activates mitochondrial function and ensures the transition of mitochondria to the active state recognized by finely tuned switches from ATP synthesis to the production of ROS, primarily superoxide and hydrogen peroxide [34,36,46].

Importantly, the activities of mitochondrial superoxide dismutase (SOD) and catalase are modulated according to the new metabolic and oxygen demands, enhancing reciprocal interactions during the oxidation of the two main mitochondrial substrates succinate and α-ketoglutarate. This prevents hypoxia by generating oxygen of metabolic origin (endogenous oxygen) and improves the general self-regulation of the metabolic system. The active state of mitochondria is an essential prerequisite for the maintenance of rapid, intensive, but low-amplitude fluctuations of ROS and their more active interactions with macromolecules in the membrane structures. As a result, not only does more branching and diverse free radical reactions occur, but also an increase in donor–acceptor interactions, which enhances the coupling of oxygen-dependent metabolic reactions and provides dynamic support for redox homeostasis (or redox homeodynamics) [16,28]. A hormetic response facilitates the development of a novel quality in redox processes, wherein subsequent hormetic influences can act as preconditioning and further train a metabolic system to improve its resilience to progressively stronger doses of stressors.

The long-term maintenance of such a beneficial functional state is determined by the coordinated interaction between key transcription factors (HIF1α, NFκB, Nrf2) and the optimal activation of pro-/antioxidant and pro-/anti-inflammatory enzymes, as well as proteins involved in proteasomal degradation and autophagy. The most significant role in maintaining redox homeostasis is played by the Keap1/Nrf2 signaling pathway as it is frequently upregulated by the electrophilic inducers capable of interacting with cysteine residues on Keap1. This interaction disrupts the repressor function of Keap1, thereby promoting the accumulation of Nrf2, its translocation to the nucleus, and the initiation of transcription of the target genes [47,48]. The electrophilic inducers of Nrf2 (hormetins) include both exogenous factors, such as moderate physical activity [49], intermittent hypoxic exposures [50], omega-3 fatty acids [51,52], polyphenols [52,53,54], and endogenous factors, namely a moderate accumulation of oxidatively modified proteins, phospholipids and bioactive lipids in cells [39,55,56].

Activation of the Nrf2 pathway by phytonutrients such as quercetin, sulforaphane, resveratrol etc. has been recognized as a promising therapeutic strategy supporting healthy longevity [42]. There is ongoing research into the development of new drugs that disrupt the protein–protein interactions between Keap1 and Nrf2, aiming to induce a hormetic response [57]. At the same time, optimal modulation of the intensity of free radical flow remains challenging particularly due to the lack of reliable and accessible biomarkers allowing monitoring of pro-oxidant/antioxidant balance and functional metabolic reserves in the human body.

## 4. Biomarkers of Oxidative Stress and Aging

Starting from the first half of the previous century, oxidative damage to the macromolecules was measured based on the levels of products of lipid peroxidation, such as diene conjugates, malondialdehyde, Schiff bases, and/or the activities of the main antioxidant enzymes such as catalase, superoxidase, and glutathione peroxidase in the biological fluids [34,58,59]. Subsequently, methodological developments allowed the measurements of many other products of oxidative destruction and nonenzymatic components of the antioxidant defense, which significantly expanded the list of the biochemical parameters characterizing the activity of the redox reactions [60,61]. Inflammation-related biomarkers such as C-reactive protein (CRP), serum amyloid A, calprotectin, cystatin C, interleukin (IL)-6, IL-8, IL-10, markers of tryptophan metabolism, and epigenetic markers (e.g., DNA Methylation Measurement) were suggested to be used to monitor aging and to predict morbidity and mortality [62,63]. The general criticism of oxidative stress biomarkers is that they do not reflect the dynamic nature of redox reactions, their intensity, capacities, and coupling, which are important determinants of the outcome of oxidative stimulation and the depth of oxidative stress [21,64,65,66]. Moreover, based on the values and changes in biochemical markers during the correction of oxidative damage, it is virtually impossible to assess the effectiveness of oxygen deficit compensation in mitochondria through the activation of endogenous antioxidant defenses [50,67]. It becomes increasingly clear that for the complex assessment of stress resilience, traditional biochemical biomarkers should be complemented with other measurements that provide complex information about the organism’s functionality. At present, multiple parameters that meet the definition of aging biomarkers were proposed and the categorization of biomarkers into molecular, physiological, histological, and radiographic is increasingly accepted [68]. Special attention is devoted to the development of digital biomarkers of aging due to the widespread use of wearables.

## 5. Heart Rate Variability as a Tool to Monitor Stress Responses

Over the past decades, the analysis of heart rate variability (HRV) has been increasingly used to assess functional metabolic potential and stress resilience [69,70,71,72]. HRV can be measured with various analytical approaches and allows estimation of the activity of the different regulatory components involved in shaping the heart rhythm. Aging is known to contribute to the reduction in HRV while age-adjusted HRV values were shown to correlate with all-cause mortality [73,74,75]. A significant interest in HRV and its broader implementation in sports, lifestyle coaching, and medicine were facilitated by the development of guidelines on HRV measurement, interpretation, and clinical use [76]. More recently, an increased accessibility of wearables sparked interest in HRV as those devices commonly utilize HRV values to estimate the capacity of the regulatory systems and their ability to respond to various challenges [77]. The most commonly used HRV indexes are derived from time domain and frequency domain analyses. HRV norms and metrics were reviewed elsewhere [78,79]. This review focuses primarily on approaches to HRV interpretation, aiming to differentiate between adaptive and maladaptive responses to stress and aging.

Our previous studies have shown that the amplitude of VLF, LF, and HF oscillations is closely related to the efficacy of aerobic metabolism (Table 1), including the energy function of the mitochondria [29,50,60,71]. The HRV parameters that can be used to characterize the functional metabolic reserve and stress resilience are summarized in Table 1. They include the values of total power (TP), which reflects the general activity of neurohumoral regulation. Higher baseline values of TP are usually associated with a more variable heart rhythm and better stress resilience. Importantly, a change in TP in response to a challenge (e.g., an orthostatic test) provides complementary information about the efficacy of functional and metabolic reserves. The values of baseline TP should always be viewed in connection with the contributing spectral components. For the short-term ECG recordings of HRV analysis, those include the following: very low frequency (VLF, 0.0033–0.04 Hz) power, which is usually associated with the general activity of the neurohumoral regulation, thermoregulation and cerebral ergotropic influences; low frequency (LF) power, which is in the range between 0.04 Hz and 0.15 Hz, and characterizes predominantly sympathetic activity; and high frequently (HF) power, which is in the range between 0.15 Hz and 0.4 Hz, and is mostly defined by the vagal tone [69].

Additionally, the LF/HF ratio which reflects the balance between sympathetic and parasympathetic control over the heart rate is frequently taken into account when monitoring the response to various stressors [23,76]. However, this parameter is controversial as its shifts do not reflect the absolute changes in the sympathetic or parasympathetic activity and should be viewed together with the absolute values of HF and LF power.

The outcome of any hormetic stimulation is determined not only by the duration and intensity of the intervention but also by the compensatory capacities of the functional systems which prevent development of the maladaptation. A mild pro-oxidant situation which is regarded as a mechanism of eustress and/or hormetic response, and which is almost universally involved in anti-aging, can be seen from the values of TP both in a supine position and during an orthostatic test, as well as specific distribution of the spectral components [60,71,78]. We believe that in an efficient HRV spectral structure, a percentage contribution of VLF components should not exceed 15–20%, while for the LF and HF components, those contributions should be 35–40% and 45–55%, respectively [60,71]. Moreover, efficient coupling of the catabolic and anabolic phases of metabolism can be seen from a specific presentation of a spectrogram, namely clustering of the highest amplitude of the LF and HF powers around 0.15–0.2 Hz. This pattern has been related to the efficient reciprocal interactions between sympathetic and parasympathetic nervous systems that contribute to a sustained hormetic response and trigger mechanisms of anti-aging [60].

**Figure 1:**
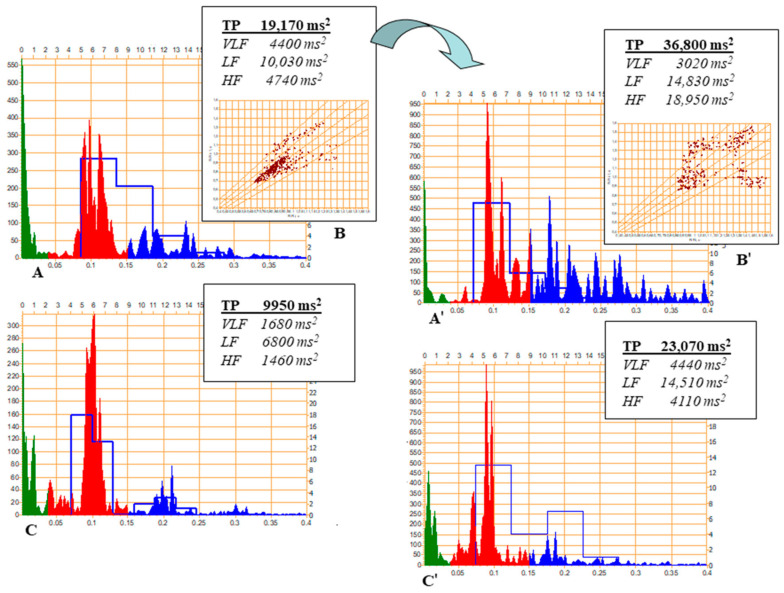
An example of heart rate variability (HRV) monitoring in an elite athlete before and after the two-week supplementation with oil derived from the seeds of *Amaranthus cruentus*. The presentation of the spectrogram (**A**) and scatter plot (**B**) in a supine position, and the spectrogram in an orthostatic position (**C**) before and after (correspondingly (**A’**–**C’**) supplementation. A significant increase in TP due to activation of the autonomic nervous system (LF and, especially, HF) is associated with the reduction (optimization) of central regulatory influences (decrease in VLF) in a supine position. Note also an “expansion of regulatory area” located around 0.15–0.2 Hz at the scatter plot A’, which may be seen as a sign of developing a hormetic response that is also linked to mechanisms of anti-aging (adapted from [80]). Note: HF—high frequency (blue color on the spectrogram); LF—low frequency (red color on the spectrogram); TP—total power; VLF—very low frequency (green color on the spectrogram). Spectrogram on the *x*-axis: top—breathing rate, 1/min; bottom—frequency, Hz; on the *y*-axis: left—amplitude, ms^2^/Hz × 1000; right—number of breathing cycles.

Figure 1 presents an example of an efficient stimulation of the functional metabolic reserves and improvement of HRV indices after a two-week intake of the oil derived from the seeds of amaranth by an elite athlete [71]. The amaranth oil has a high content of linoleic acid and includes such minor components as squalene, tocotrienols, tocopherols, and phytosterols, which ensure its effect on the redox metabolism [71]. An observed increase in the values of TP in the supine position, improved spectral structure, clustering of HF and LF at around 0.15–0.2 Hz on the spectrogram, and capacity to maintain the relatively high values of TP in orthostasis (Figure 1) are suggestive of the beneficial modulation of the functional metabolic reserves as a result of this intervention [71].

## 6. Proposed Mechanisms Linking HRV and Redox Metabolism and Inflammation

The evidence supporting the links between oxidative metabolism and the activity of autonomic regulation, which is commonly used in interpreting HRV, is still limited. However, several mechanisms may play a role in these bidirectional interactions. Firstly, the data from in vitro studies involving isolated organs and tissues clearly shows the impact of oxidative stress on the reactivity of membrane receptors involved in the autonomic regulation [81,82]. The experiments in isolated rat heart, lung, intestinal tissues, and immune cells demonstrated that oxidative damage to the membranous structures caused by excessive lipid peroxidation impairs the functionality of the adrenergic and cholinergic receptors [82,83,84]. The malfunction of the adrenergic receptors is further exacerbated by treating the animals with antioxidant-deficient diets [82]. Mechanistically, changes in the membrane fluidity due to excessive lipid peroxidation, direct damage to receptor structures, and dysfunctional G protein-coupled receptor—cyclic adenosis monophosphate (GPCR-cAMP) signaling were suggested to play a role in impaired responses to beta-adrenoreceptor-mediated responses of the heart [83]. This also applies to the pacemaker cells in the sinus node whose functionality is largely defined by receptor integrity with degradation and internalization of the receptors adding additional strain on the regulatory homeostatic mechanisms, which can lead to an increase in the activity of the cerebral ergotropic regulation. In studies with isolated organs (rat tracheal strips, intestine, canine heart), an excessive amount of oxidants was shown to reduce muscarinic cholinergic receptor-mediated contraction of the smooth muscles [82] and reduce maximal receptor binding to [3H]quinuclidinyl benzilate (QNB) [85], indicating that peroxidation of membrane lipids may indirectly lead to a decreased muscarinic receptor function.

Another mechanism that can potentially explain the links between HRV and oxidative metabolism involves inflammation. Oxidative stress and accumulation of the products of oxidative damage were shown to facilitate inflammatory responses involved in chronic diseases and aging [86,87,88]. In the human body, the autonomic nervous system acts as an allostatic regulator of the immune response [89,90]. The pro-inflammatory immune response was demonstrated to contribute to sympathetic hyperactivity promoting the persistence of chronic local and systemic low-grade inflammation [91]. Evidence from experimental models offers more insight into the relevant pathobiological mechanisms [91]. For example, in a rodent model of inflammatory arthritis, it was shown that maladaptive changes in β2-adrenergic receptor signaling in the spleen with a transition from protein kinase A (PKA) to mitogen-activated protein kinase (MAPK) pathways can drive inflammation and contribute to the disease progression [92]. Moreover, the therapeutic potential of vagal stimulation in the management of various inflammatory diseases has been well recognized due to its ability to inhibit the production of pro-inflammatory cytokines, change the profile of the circulating immune cells, and modulate the generation of ROS by macrophages (so-called anti-inflammatory cholinergic reflex) [90,93,94]. Thus, both upregulation of the sympathetic response and reduced vagal activity were suggested to contribute to the persistence of chronic low-grade inflammation.

An autonomic dysregulation measured by HRV was confirmed to be present in many immune-mediated inflammatory diseases [95]. The meta-analysis involving 51 clinical studies showed that levels of inflammatory biomarkers correlate with values of HRV. Particularly, the time-domain parameter, the standard deviation of R-R intervals (SDNN), and HF power showed the strongest and most robust associations [95], substantiating the potential of HRV as a valuable tool to monitor the activity of the neurophysiological pathway responsible for regulating inflammatory processes in humans.

In addition to its regulatory influence on the immune response, the autonomic nervous system has been bidirectionally connected to the functionality of mitochondria. The autonomic neurotransmitters, epinephrine and acetylcholine, can also serve as activators of oxidation of the main mitochondrial substrates succinate and α-ketoglutarate, which are involved in the regulation of the ATP potential (ATP/ADP ratio) [36,37,96,97]. In the model involving glass-adhered lymphocytes in blood smear, which provides ex vivo stable preservation of the in vivo mitochondrial network, adrenergic stimulation in vivo caused two- to eightfold increase in succinate dehydrogenase activity, while the activity of alpha-ketoglutarate dehydrogenase changed reciprocally [98]. The increase in cholinergic regulation was related to higher oxidation of α-ketoglutarate and selective activation of α-ketoglutarate dehydrogenase [98,99]. Those model studies suggest the regulatory effect of the autonomic neurotransmitters on the functionality of mitochondria.

In another model involving LPS-activated macrophages, a buildup of succinate resulted in increased production of ROS, which was hypothesized to be involved in stabilization of HIF-1α and expression of the pro-inflammatory IL-1β [37]. Significantly increased activity of the sympathetic nervous system causes increased fluctuation of the intracellular Ca^2+^, which strains the mitochondria and contributes to reduced ATP generation, excessive production of ROS, and accumulation of products of oxidative damage [100]. Thus, autonomic regulation might have a modulatory effect on the ROS production by mitochondria, which in turn is an important signaling organelle orchestrating a variety of (patho-)physiological cellular responses [37].

Under physiological conditions including healthy longevity, the production of superoxide, hydrogen peroxide, and hydroxyl radicals, which are formed as a result of one-, two-, or three-electron reduction in the molecular oxygen in mitochondria, facilitates dynamic fluctuations in redox homeostasis by upregulating antioxidant defense and further promoting free radical reactions. Superoxide and hydrogen peroxide can also serve as a source of molecular oxygen in a cell upon being transformed by superoxide dismutase and catalase. An excessive production of mitochondrial ROS can contribute to persistence of inflammation and accelerated aging [13,37]. Furthermore, a stimulant role of ROS on sympathetic activity resulting in an increase in blood pressure was demonstrated in an in vivo study in rodents [101].

Another mechanism potentially linking HRV to redox metabolism involves sulfur-containing compounds which were proposed to act as oxygen sensors [102,103]. Mitochondria play an important role in the metabolizing of sulfur-containing compounds to H_2_S, or thiosulfates, sulfites, and sulfates (H_2_S-S_2_O_3_^(2−)^-SO_3_^(2−)^-SO_4_^(2−)^) depending on the activity of the oxygen-dependent enzymes [102,104]. Exogenous administration of H_2_S was shown to elicit physiological responses similar to hypoxia, including the upregulation of the sympathetic nervous system [102]. The modulatory effect of H_2_S on autonomic balance was shown to be achieved via modification of baroreflex afferent function, which contributes to the regulation of blood pressure responses [105]. The effects of H_2_S on cardiovascular function, in general, are mediated by activation of the ATP-dependent K^+^ channels (K_ATP_) [106], which causes hyperpolarization of vascular smooth muscle cells and promotes vasodilation by reducing voltage-gated Ca^2+^ influx. Another physiologically relevant target of H_2_S are large-conductance calcium- and voltage-activated K^+^ (BKCa) channels [106,107]. The reports illustrating the mechanisms involved in the regulation of BKCa channels by H_2_S are conflicting; however, their involvement in the stimulation of the hypoxic excitation of the carotid body, as well as the mechanisms involved in the protection of ischemia–reperfusion injury are supported by existing evidence [106]. Under the condition of oxidative stress, which is usually accompanied by the development of hypoxia, H_2_S, and reactive sulfur species act as oxygen sensors and modulate the activity of cardiovascular response via changes in autonomic regulation [102]. The BKCa channels play a role in modulating the electrical activity of the sinus node, particularly via interference with diastolic depolarization and pacemaker activity of the cardiomyocytes [60]. Therefore, the regulation of BKCa by H_2_S in pacemaker cells of the sinus node [108] might represent a direct mechanism linking redox homeostasis with the regulation of the heart rate, which can be assessed by measurement of HRV.

**Figure 2:**
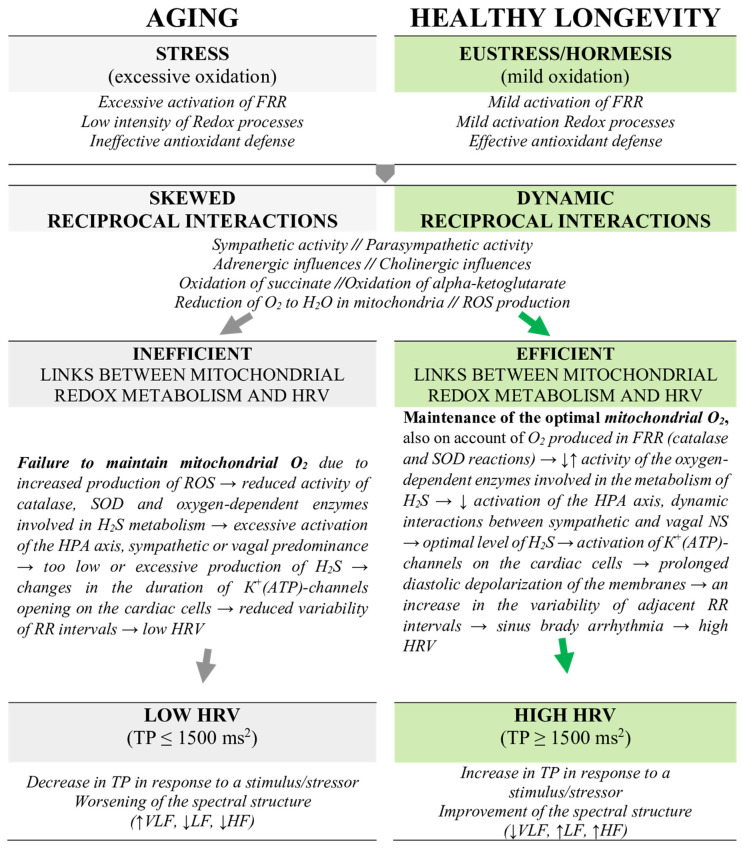
Metabolic background and mechanisms involved in aging versus healthy longevity. The initiation of aging or anti-aging mechanisms depends on the degree to which the intensity of the stimulation aligns with the coping capacities of the functional metabolic reserves. In general, anti-aging processes are initiated by mild stimulation of the redox reaction and the transition of mitochondria to an active energy state without imposing excessive strain on the cardiovascular system, which is reflected by HRV. The changes in HRV parameters can help to monitor and adjust the dose for various interventions (e.g., intake of dietary ingredients, physical activity, activities involving creativity, learning, and evoking positive emotions), helping to achieve optimal health benefits. FRR—free radical reactions; HRV—heart rate variability; HPA—hypothalamic–pituitary–adrenal; HF—high frequency; LF—low frequency; NS—nervous system; ROS—reactive oxygen species; SOD—superoxide dismutase; TP—total power; VLF—very low frequency.

We hypothesize that this mechanism can be related to the development and maintenance of sinus bradyarrhythmia, which is characterized by an increased variation in the duration of the consecutive RR intervals. Those are recognized to be predominantly determined by vagal regulation and are closely linked to the activity of the HF component in the spectral structure of HRV. The spectral profile of HRV characterized by increased activity of HF and LF components with reduced contribution of the VLF is supported by the efficient functionality of the energy metabolism, characterized by the higher capacity to maintain the mitochondrial O_2_/ROS balance as well as the balances between H_2_S and thiosulfates (sulfites, sulfates, Figure 2). Significant shifts in those balances trigger excessive free radical destruction of macromolecules and promote oxidative stress, ultimately leading to aging. On the contrary, fine-tuned reciprocal redox interactions that support mild pro-oxidant activity are crucial for maintaining mitohormesis, which is an important determinant of the eustress reaction at the level of an organism [43,44,60]. 

The support of the anti-aging mechanisms/hormetic responses requires stimulation of FRR that exceeds the inhibitory capacity of the direct antioxidants. Such prolonged pro-oxidant predominance requires close monitoring of the intensity of the FRR which must be kept at the desirable low-to-medium-high levels. The transition of the mitochondria to an active energy state stimulates redox metabolism, prevents excessive destruction of the cellular components, and promotes the development of the hormetic response [3,43,44,109]. Aging is associated with the persistence of signs of oxidative stress, which the parameters of HRV have been shown to decrease with advancing age [69,110,111,112,113]. The factors contributing to cellular senescence include accumulation of oxidatively damaged macromolecules, a substantial predominance of ROS production over the capacity of the endogenous antioxidant defense, hypoxia, loss of effective lysosomal autophagy, and impaired redox signaling, which altogether compromise the cellular metabolism [28,114]. It is postulated that described biochemical changes lead to the transformation of cells into a quiescent state with a subsequent decline in subcellular regeneration processes and ultimate progression toward aging [114]. A potential reversal from this state could involve the reactivation of cells from quiescence to metabolic activity and the restoration of regenerative processes [114]. In our opinion, this aligns closely with changes caused by mild-to-moderate stimulation of redox reactions, which include gradual but constant elimination of products of oxidative damage and the regeneration of cells and tissues regulated among others by the autonomic nervous system. The efficiency of reciprocal switching between the sympathetic and parasympathetic components is reflected on the electrocardiogram as marked alternations in the duration of adjacent R-R intervals, which forms the basis for the higher HRV.

## 7. Conclusions

Nowadays, the free radical theory of aging can be complemented by the free radical theory of longevity, which offers a new paradigm to support healthy aging. It is increasingly recognized that mild-to-moderate upregulation in the production of free radicals plays an important physiological role in cellular signaling and can trigger the mechanisms of antioxidant defense, supporting an adaptive response to various stressors. This so-called hormetic response results in the improvement of the functional metabolic reserves and is related to healthy aging as well as to the effects of anti-aging interventions. On the other hand, excessive production of free radicals contributes to the development of oxidative stress and leads to aging. Therefore, the search for biomarkers that would allow efficient assessment of redox homeostasis is of great importance in the monitoring of healthy aging.

We hypothesize that HRV, which measures the changes in the duration between the adjacent RR intervals, is largely defined by the activity of the redox homeostasis and, therefore, can be used as a biomarker of aging. Such reasoning is based on several lines of experimental evidence suggesting mechanistic links between the autonomic regulation and oxidative load. In this paper, the modulatory effect of well-characterized oxygen sensor H_2_S on cardiovascular function and pacemaker activity of the sinus node, the studies on the direct effects of free radicals on the functionality of adrenergic and cholinergic receptors, and demonstrated bidirectional interactions between the activity of the autonomic nervous system and immune response were introduced to support the hypothesis about the close interactions between the production of ROS and autonomic regulation and, thus, HRV. At the same time, further studies are needed to improve our understanding of the crosstalk between mitochondrial function and autonomic regulation.

Based on our previous studies, the practical approach to interpretation of HRV was proposed. It identifies changes in several HRV indices, namely higher values of TP, which are readily maintained during the response to stressors, higher values of LF, and HF, with reduced contribution of the VLF power to the spectral structure as biomarkers of the efficient adaptation. However, those assumptions still need to be tested in clinical studies involving anti-aging interventions and be connected to other biomarkers of aging to provide a complex overview of this process and better define the role of HRV in the monitoring of aging.

## Figures and Tables

**Table 1:** Relationship between heart rate variability and stress resistance.

Functional Metabolic Reserve	HRV Parameters				Changes in Response to Challenges	TP_ortho/_ TP_sup_
	TP, ms^2^	VLF, %	LF, %	HF, %		
**Good** (preserved resistance to oxidative stress)	>3000	<20	25–35	>45	↑TP (↓ VLF, ↑↓ LF, ↑ HF)	0.5–1.5
**Moderate strain** (slightly reduced resistance to oxidative stress)	1700–3000	20–40	25–35	35–45		
**Overstrain** (low resistance to oxidative stress)	900–1500	30–65	25–35	20–30	↑↓TP (↑ VLF,↓ LF, ↓ HF)	<0.5 or >1.5
**Exhaustion** (very low resistance to oxidative stress)	<900	40–80	15–25	6–25		

Note: HF—high frequency; LF—low frequency; TP—total power; TP_ortho/_TP_sup_—a ratio of values of TP during the orthostatic test to the values in supine position; VLF—very low frequency; light green indicates beneficial responses; light grey indicates potentially hazardous responses.
